# Alpha‐synuclein fibrils amplified from multiple system atrophy and Parkinson's disease patient brain spread after intracerebral injection into mouse brain

**DOI:** 10.1111/bpa.13196

**Published:** 2023-07-24

**Authors:** Shuyu Zhang, Karina Dauer, Timo Strohäker, Lars Tatenhorst, Lucas Caldi Gomes, Simon Mayer, Byung Chul Jung, Woojin S. Kim, Seung‐Jae Lee, Stefan Becker, Friederike Liesche‐Starnecker, Markus Zweckstetter, Paul Lingor

**Affiliations:** ^1^ Clinical Department of Neurology, School of Medicine, University Hospital rechts der Isar Technical University of Munich Munich Germany; ^2^ Department of Neurology University Medical Center Göttingen Göttingen Germany; ^3^ Center for Biostructural Imaging of Neurodegeneration University Medical Center Göttingen Göttingen Germany; ^4^ German Center for Neurodegenerative Diseases (DZNE) Göttingen Germany; ^5^ Department of Biomedical Sciences, Neuroscience Research Institute, Convergence Research Center for Dementia, College of Medicine Seoul National University Seoul South Korea; ^6^ Faculty of Medicine and Health, Brain and Mind Centre and School of Medical Sciences The University of Sydney Sydney New South Wales Australia; ^7^ School of Medical Sciences University of New South Wales and Neuroscience Research Australia Randwick New South Wales Australia; ^8^ Department of NMR Based Structural Biology Max Planck Institute for Multidisciplinary Sciences Göttingen Germany; ^9^ Department of Neuropathology, Institute of Pathology, School of Medicine Technical University Munich Munich Germany; ^10^ Department of Pathology and Molecular Diagnostics, Medical Faculty University of Augsburg Augsburg Germany

**Keywords:** alpha‐synuclein, microglia, multiple system atrophy, Parkinson's disease, patient‐derived fibrils

## Abstract

Parkinson's disease (PD), multiple system atrophy (MSA), and dementia with Lewy bodies (DLB) are neurodegenerative disorders with alpha‐synuclein (α‐syn) aggregation pathology. Different strains of α‐syn with unique properties are suggested to cause distinct clinical and pathological manifestations resulting in PD, MSA, or DLB. To study individual α‐syn spreading patterns, we injected α‐syn fibrils amplified from brain homogenates of two MSA patients and two PD patients into the brains of C57BI6/J mice. Antibody staining against pS129‐α‐syn showed that α‐syn fibrils amplified from the brain homogenates of the four different patients caused different levels of α‐syn spreading. The strongest α‐syn pathology was triggered by α‐syn fibrils of one of the two MSA patients, followed by comparable pS129‐α‐syn induction by the second MSA and one PD patient material. Histological analysis using an antibody against Iba1 further showed that the formation of pS129‐α‐syn is associated with increased microglia activation. In contrast, no differences in dopaminergic neuron numbers or co‐localization of α‐syn in oligodendrocytes were observed between the different groups. Our data support the spreading of α‐syn pathology in MSA, while at the same time pointing to spreading heterogeneity between different patients potentially driven by individual patient immanent factors.

## Graphical abstract

Injection of alpha‐synuclein (α‐syn) fibrils amplified from brain homogenates of two multiple system atrophy patients and two Parkinson's disease patients into the brains of C57Bl6/J mice cause different levels of α‐syn spreading.

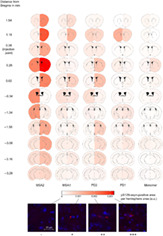

## 1 INTRODUCTION

Parkinson's disease (PD) and multiple system atrophy (MSA) are neurodegenerative movement disorders that share a long preceding non‐motor phase characterized by sleep disturbances such as insomnia, circadian rhythm disruption, and rapid‐eye‐movement sleep behavior disorder (RBD) [1]. These common clinical symptoms are explained by similarities in the pathophysiology of PD and MSA, both belonging to the family of α‐synucleinopathies. In PD, misfolded alpha‐synuclein (α‐syn) aggregates in neurons as Lewy bodies (LB), whereas in MSA the protein accumulates mainly in oligodendrocytes as glial cytoplasmatic inclusions (GCI) [2].

To reproduce the pathophysiology of these α‐synucleinopathies, several animal models have been developed based on injection of in vitro‐generated α‐syn aggregates into transgenic mice, or viral vectors inducing α‐syn overexpression [3, 4]. The injection of preformed fibrils generated from recombinant α‐syn proteins into the mouse brain is a promising animal model to study α‐synucleinopathies, as it results in a spreading pathology of phosphorylated α‐syn aggregates [5, 6, 7]. These phosphorylated α‐syn aggregates are proteinase‐K‐resistant and thioflavin‐S‐positive and colocalize with ubiquitin and p62 similar to human LBs [6].

Misfolded fibrillar α‐syn propagates itself and spreads from cell to cell in anatomically interconnected regions [8]. In addition, different α‐syn strains seed and spread aggregates to various extents [9, 10]. For example, α‐syn strains were shown to vary between PD and MSA [11]. When injected into the mouse brain, MSA strains accelerate neurodegeneration as compared to PD strains [12].

In the current study, we investigated the propagation of α‐syn in the mouse brain after injection of α‐syn fibrils amplified from homogenates of two MSA and two PD patients [13]. In addition, we quantified the number of microglia, astrocytes and neuronal cell death in the substantia nigra pars compacta (SNpc) induced by the injection of the patient material‐amplified fibrils, as well as the colocalization between pS129‐α‐syn and the oligodendrocytic marker 2′,3′‐cyclic nucleotide‐3′‐phosphodiesterase (CNPase).

## 2 MATERIALS AND METHODS

**FIGURE 1:**
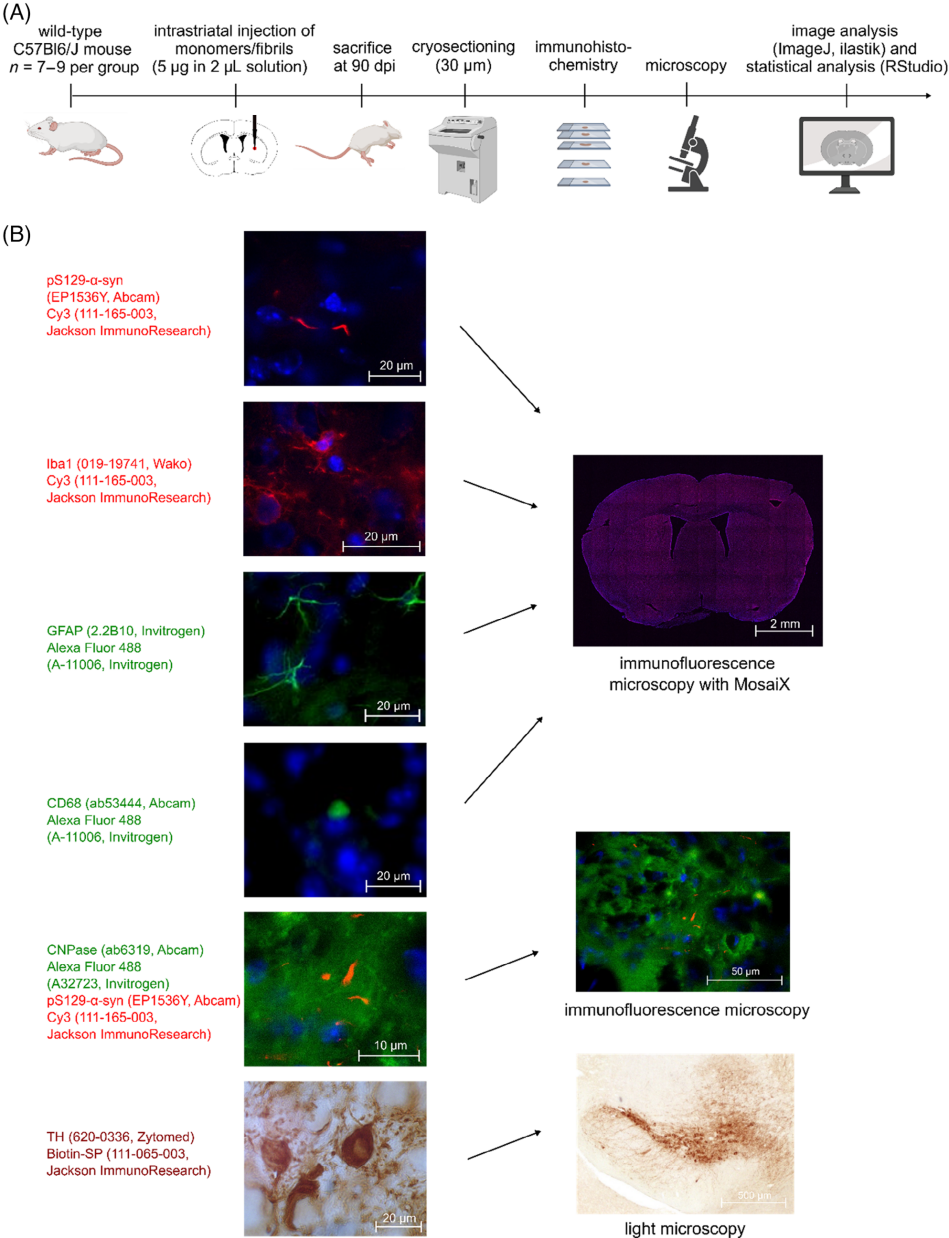
(A) Workflow of the study consisting of patient‐derived fibril injection, brain sectioning, immunohistochemical staining, microscopical imaging, image processing, and statistical analysis. (B) Immunohistochemistry: antibodies used for immunohistochemical analyses are indicated next to representative images. CNPase, 2′,3′‐cyclic nucleotide‐3′‐phosphodiesterase; dpi, days postinjection; GFAP, glial fibrillary acidic protein; TH, tyrosine hydroxylase.

The workflow of the study as well as exemplary images for immunohistochemistry stainings are shown in Figure 1.

### 2.1 Amplification of α‐syn fibrils from protein misfolding cyclic amplification products and generation of α‐syn monomers

Following ethics approval from the University of New South Wales Human Research Ethics Committee (approval number: HC16568), brain tissues were received from the Sydney Brain Bank at Neuroscience Research Australia which is supported by The University of New South Wales and Neuroscience Research Australia. α‐syn aggregates used in the current study had been previously amplified from brain extracts of patients pathologically confirmed with PD and MSA using protein misfolding cyclic amplification (PMCA; Table 1) [13]. To obtain sufficient quantities for structural analysis, PMCA‐amplified amyloid fibrils were used in a second step to seed recombinant α‐syn [13].

**TABLE 1:** Clinical characteristics of patient material and monomer preparations used in this study [13].

Group	Source	Disease duration (years)	Age at death	Sex	Cause of death	Number of animals injected
PD	PD1	7	79	M	Acute myocardial infarction	8
	PD2	8	82	F	Pneumonia	7
MSA	MSA1	7	82	M	Cardiorespiratory failure	7
	MSA2	6	71	F	Hypostatic pneumonia	8
α‐syn monomer	Recombinantly expressed from *Escherichia coli*	n.a.				8

*Note*: The number of animals injected with each fibril/monomer preparation is given additionally.

Abbreviations: MSA, multiple system atrophy; PD, Parkinson's disease; α‐syn, alpha‐synuclein.

Basic demographic information on brain samples used for α‐syn fibril amplification is given in Table 1.

N‐terminally acetylated α‐syn was obtained by co‐transfection of *Escherichia coli* BL21 (DE3) cells with pT7‐7 plasmid encoding for human α‐syn (kindly provided by the Lansbury Laboratory, Harvard Medical School, Cambridge, MA) and *Schizosaccharomyces pombe* NatB acetylase complex [14] using pNatB plasmid (pACYCduet‐naa20‐naa25, Addgene, #53613, kindly provided by Dan Mulvihill). Protein expression and purification were performed as described [15].

α‐Syn fibrils were prepared at 37°C from monomeric, N‐terminally acetylated α‐syn taken from the supernatant of freshly thawed α‐syn on ice after ultracentrifugation (Beckman Coulter Optima MAX‐XP using a TLA 100.3 rotor with a rotor speed of 55,000 rpm). Then, 0.5% (w/w) PMCA product was added to 250 μM αSyn stock solution (50 mM 4‐(2‐hydroxyethyl)‐1‐piperazineethanesulfonic acid [HEPES], 100 mM NaCl, pH 7.4, 0.02% NaN_3_) and initially water bath sonicated for 10 min. This mixture was aggregated under quiescent conditions in 1.5‐mL Eppendorf cups in a ThermoScientific Heratherm incubator.

### 2.2 Animal experiments

Wild‐type C57BI6/J male mice (RRID: IMSR_JAX:000664) were purchased from Charles River (Wilmington, USA) and housed in the Central Animal Care Unit of the University Medical Center Göttingen, Germany. The animals were treated according to the EU Directive 2010/63/EU for animal experiments and the regulations of the local animal research council as well as the legislation of the State of Lower Saxony, Germany (ethics approval number: 33.9‐42502‐04‐15/1982) in an exploratory study which has received approval from the institutional ethics committee. Mice were housed in individually ventilated cages (Tecniplast) with standard ad libitum food, water, and a 12‐h dark/light cycle. No randomization was performed to allocate subjects to the study, no exclusion criteria were predetermined. Altogether 42 male mice were used in this study. The average body weight of all mice was 25.4 g. This study was not preregistered.

Injections were performed as previously described [5]. At the age of 12 weeks, mice were injected with α‐syn monomers or fibrils into the right striatum (anteroposterior axis +0.4 mm; mediolateral axis −1.8 mm; dorsoventral axis −3.5 mm relative to Bregma). Both monomers and fibrils were diluted in 50 mM HEPES buffer, and 5 μg were finally injected in 2 μL final volume. Before the injection, α‐syn fibrils were sonicated for 30 s in one cycle at 10% power (Bandelin Sono Plus, Bandelin, Berlin, Germany). Animals were divided into five groups: one control group (monomeric α‐syn *n* = 9) and four treatment groups (PD1 *n* = 8, PD2 *n* = 7, MSA1 *n* = 7, MSA2 *n* = 8). Three animals died because of complications of surgery.

Two days before and 2 days after the injection of α‐syn, the animals received an analgesic treatment with Metamizole (1.5 mg/mL) in drinking water. For the injection, mice were anesthetized by intraperitoneal injection of ketamine (150 mg/kg body weight) and xylazine (10 mg/kg body weight) and fixed in a stereotactic frame. Eyes were protected by using an eye ointment. After incision and trepanation, the solution was injected at an injection rate of 500 nL/min using a glass capillary of 100 μm diameter and a microinjector (Micro 4, World Precision Instruments, Friedberg, Germany). To prevent reflux, the injection capillary was left in place for 4 min before it was slowly removed, and the incision was closed with tissue glue (DermaBond, Ethicon, Raritan, USA). After surgery, mice were placed on a warming pad until wake‐up and then returned to their home cage.

Finally, the animals were sacrificed at 90 days postinjection by a lethal intraperitoneal injection of ketamine (300 mg/kg body weight) and xylazine (15 mg/kg body weight) solution. Under deep anesthesia, mice were transcardially perfused with 50‐mL ice‐cold phosphate‐buffered saline (PBS) within 5 min followed by 50 mL of 4% paraformaldehyde (PFA, Applichem, Darmstadt, Germany) at pH 7.4 within 5 min. Then, the mouse brains were removed, fixed in 4% PFA/PBS for 24 h at 4°C and then transferred into 30% sucrose in PBS for cryopreservation before freezing at −80°C.

### 2.3 Cryosectioning

Complete coronal sections of all 39 brains were prepared at a thickness of 30 μm at −20°C using a Leica CM3050S Cryostat (Leica, Wetzlar, Germany). Sections were mounted on Superfrost slides and stored at −80°C until further processing. Six sectional planes were selected based on important anatomical hallmarks for immunohistochemical staining and defined by their distance to Bregma: + 1.54 mm (striatum), +0.38 mm (striatum, location of injection point), +0.02 mm (striatum), −1.58 mm (hippocampus), −3.08 mm (substantia nigra), −3.28 mm (substantia nigra), according to coordinates given in the Paxinos Mouse Brain Atlas [16]. These six regions were stained for pS129‐α‐syn and Iba1. Additional five regions (+1.18 mm, +0.26 mm, −0.34 mm, −1.34 mm, −3.16 mm) were also stained for pS129‐α‐syn, Iba1, CD68, and glial fibrillary acidic protein (GFAP).

### 2.4 Immunohistochemistry

Immunohistochemistry was performed according to adapted protocols as previously described [5]. First, mounted sections were dried at 21°C for 45 min. They were rehydrated in PBS for 15 min and then steamed at 80°C in 10 mM citrate buffer (pH = 6.0) for 30 min. After 2 × 5 min of washing in PBS, sections were incubated in 25 mM glycine in PBS and washed again 2 × 5 min in PBS. Sections were then incubated in a blocking solution (5% normal goat serum [NGS], 5% bovine serum albumin, 0.3% TritonX, 25 mM Glycine in PBS) for 60 min and washed for 5 min in PBS. The anti‐pS129 α‐syn antibody (EP1536Y, #ab51253, Abcam, Cambridge, UK) was diluted at 1:500, the anti‐Iba1 antibody (019‐19741, Fujifilm Wako, Osaka, Japan) was diluted at 1:300, the anti‐CD68 antibody (FA‐11, ab53444, Abcam) was diluted at 1:500, the anti‐CNPase antibody (ab6319, Abcam) was diluted at 1:150, and the anti‐GFAP antibody (13‐0300, Invitrogen, Waltham, MA, USA) was diluted at 1:250 in blocking solution. For the additional five regions, we used another anti‐Iba1 antibody diluted at 1:250 (011‐27991, Fujifilm Wako). Then, sections were incubated with the primary antibodies in a moist chamber overnight at 4°C. The next day, the sections were washed 3 × 10 min in PBS and incubated with the secondary antibody in a dark chamber for 60 min at 21°C. Cy3 was used for the anti‐α‐syn and the anti‐Iba1 staining (111‐165‐003, Jackson ImmunoResearch, West Grove, PA, USA, dilution 1:250), Alexa Fluor 647 for the anti‐goat anti‐Iba1 antibody (A32849, Invitrogen, dilution 1:250), Alexa Fluor 488 (A‐11006, Invitrogen, dilution 1:250) for the anti‐CD68 and the anti‐GFAP staining and another Alexa Fluor 488 (A32723, Invitrogen, dilution 1:150) for the anti‐CNPase staining. Following a washing step with PBS for 3 × 10 min, the sections were incubated in 1 μg/mL 4′,6‐diamidino‐2‐phenylindole (DAPI; Thermo Fisher Scientific, Waltham, MA, USA) in PBS for 5 min and washed with PBS for 2 × 10 min. Finally, the sections were dried at 37°C in an incubator for 10 min and mounted with EcoMount (Biocare Medical, Pacheco, CA, USA).

For each brain, three sections of the substantia nigra (SN) at around Bregma +3.00, +3.15, and +3.30 mm were stained for tyrosine hydroxylase (TH) to visualize the dopaminergic neurons. These sections were first dried for 45 min, rehydrated in PBS for 10 min, and then incubated in PBS with 40% methanol and 1% H_2_O_2_. Following a washing step with PBS for 3 × 5 min, the sections were blocked for 60 min using a solution consisting of 5% NGS (Cedarlane, Burlington, ON, Canada) and 0.05% Triton X‐100 (AppliChem) in PBS. The blocking solution was decanted, and the primary anti‐TH antibody (620‐0336, Zytomed, Bargteheide, Germany) diluted 1:1000 in 2.5% NGS and 0.025% TritonX in PBS was added to the sections. After an incubation period of 24 h at 4°C in a moist chamber, the slides were washed 3 × 5 min and incubated at 21°C for 2 h with the biotinylated secondary antibody (111‐065‐003, Jackson ImmunoResearch, 1:200, diluted in 2% NGS in PBS). The tissue was washed for 3 × 5 min and then incubated in the Vectastain ABC Peroxidase Kit for 2 h to enhance the signal before it was washed again. The sections were incubated for 8 min in the Vector 3,3′‐diaminobenzidine (DAB) substrate kit (two drops DAB reagent 1, four drops DAB reagent 2, two drops DAB Reagent 3), which visualizes the TH‐staining with DAB. Next, the reaction was stopped in distilled water for 5 min and the slides were washed one last time in PBS for 2 × 5 min. Finally, the slides were mounted with Entellan (Merck Millipore, Burlington, MA, USA) and dried at 21°C.

### 2.5 Microscopy and image analysis

Stained sections were imaged using the Axio Observer Z1 (Zeiss, Oberkochen, Germany). For immunofluorescence imaging, we used the MosaiX function of the AxioVision software with 10x magnification to generate whole‐brain sections. The anti‐CNPase anti‐α‐syn stainings were imaged with 63× magnification by meandering through the entire section. The SN sections were imaged at 5× magnification for each hemisphere separately.

The images were loaded into ImageJ/Fiji (version 1.53c) [17] to quantify the amount of pS129 α‐syn‐positive signal. Regions of interest (ROIs) were defined manually by outlining each hemisphere in the merged image of the red (pS129 α‐syn) and blue (DAPI) channel. The ROI areas were quantified, and the channels were split. In the red channel, the background was subtracted after the definition of a manual threshold. To exclude bias because of variability in manual thresholding, we performed a Kruskal–Wallis rank sum test indicating no significant difference between the thresholds of the five groups. With the *analyze particles* function, the area of pS129 α‐syn‐positive aggregates was calculated for each hemisphere. For each brain, the signal‐positive area was divided by the total area of the corresponding hemisphere.

For the anti‐CNPase/anti‐α‐syn double immunofluorescence staining, we analyzed sections at +0.20 mm from Bregma using the JACoP plugin in ImageJ/Fiji [18] and calculated Pearson's coefficient and Manders' coefficient for the original and the thresholded image.

For the anti‐Iba1‐staining and the GFAP‐staining, six circular ROIs were defined for each brain section. The background was subtracted, and an automatic threshold was set. The *analyze particles* function was used to quantify the area of Iba1‐positive cells covering the total area of each ROI. The anti‐CD68‐staining was analyzed in a similar way using one circular ROI in the striatum.

The anti‐TH DAB staining was analyzed using the software ilastik (version 1.3.3post3) [19] and ImageJ/Fiji [17]. First, ROIs were defined manually in ImageJ/Fiji by outlining the SNpc. Using the pixel classification function in ilastik, a mask for TH‐positive cells was created and these were quantified in ImageJ/Fiji.

### 2.6 Statistical analysis

RStudio (version 1.4.1106) was used for statistical analysis. Data were tested for normality using the Shapiro–Wilks test. For normally distributed data, as in the anti‐TH staining, we performed an analysis of variance (ANOVA) to compare the different groups and a paired *t*‐test to compare both hemispheres. The data on the α‐syn‐staining, the Iba1‐staining, the GFAP‐staining, and the CNPase‐staining were not normally distributed; therefore, we performed a Kruskal–Wallis rank sum test with a post hoc Dunn's test.

## 3 RESULTS

### 3.1 Strain influences on α‐syn spreading

**FIGURE 2:**
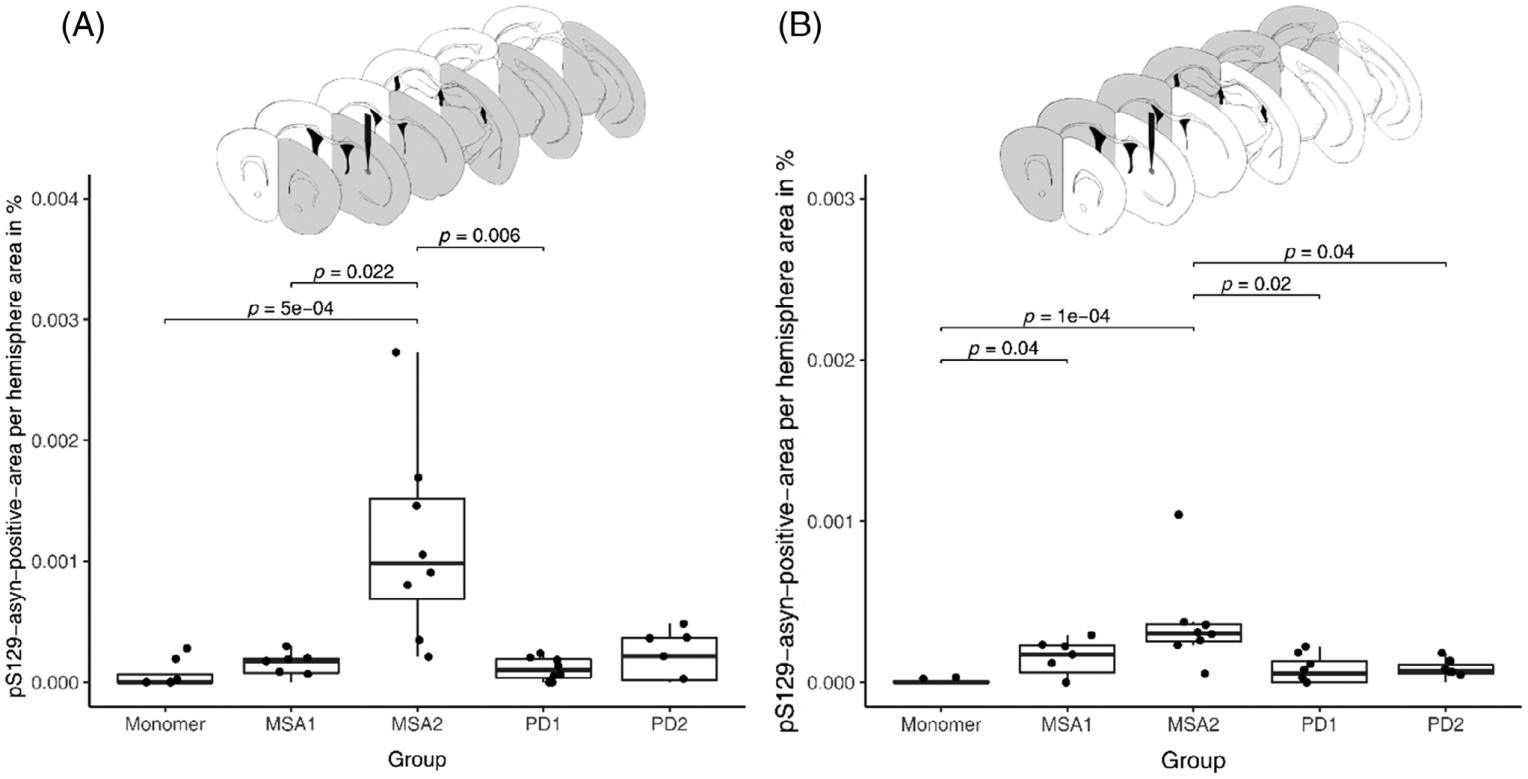
Quantification of pS129‐positive α‐syn aggregates in mouse brains injected with different brain‐derived α‐syn strains. Area of pS129‐positive α‐syn aggregates per hemisphere area in the injected (A) and non‐injected (B) hemispheres. A *p*‐value <0.05 was considered as significant according to the Kruskal–Wallis test and Dunn's test.

After the injection of α‐syn monomers or fibrils, we quantified the area of α‐syn aggregates for each brain hemisphere (Figure 2). Overall, we found significant differences in the amount of pS129‐positive signal between the five groups injected with α‐syn fibrils of PD1, PD2, MSA1, MSA2, or monomeric α‐syn as control. α‐syn monomers are commonly used as non‐aggregated controls in α‐syn fibril spreading experiments [5, 10, 20, 21, 22, 23]. For the injected (right) hemisphere, brains from the MSA2 group had the highest abundance of pS129‐α‐syn‐positive aggregates, followed by PD2 and MSA1. As the data were not normally distributed, we performed a Kruskal–Wallis rank sum test (Kruskal–Wallis chi‐squared = 18.8, d.f. = 4, *p*‐value = 0.0009 for all regions of the injected hemisphere), followed by a post hoc Dunn's test with Benjamini–Hochberg *p*‐value correction. For the injected hemisphere, the pairwise comparison indicated that there were significant differences between PD1 and MSA2 (*p* = 0.0058), MSA1 and MSA2 (*p* = 0.0222), and MSA2 and control (*p* = 0.0004; Figure 2A).

For the non‐injected (left) hemispheres, brains injected with MSA2‐derived α‐syn fibrils also showed the highest abundance of α‐syn aggregates. The Kruskal–Wallis rank sum test implied significant differences between the five groups (Kruskal–Wallis chi‐squared = 20.4, d.f. = 4, *p*‐value = 0.0004 for all the regions of the non‐injected hemisphere) and Dunn's test with Benjamini–Hochberg *p*‐value correction revealed significant differences between brains injected with α‐syn fibrils from PD1 and MSA2 (*p* = 0.0166), PD2 and MSA2 (*p* = 0.0402), MSA1 and control (*p* = 0.0402), as well as MSA2 and control (*p* = 0.0001; Figure 2B).

**FIGURE 3:**
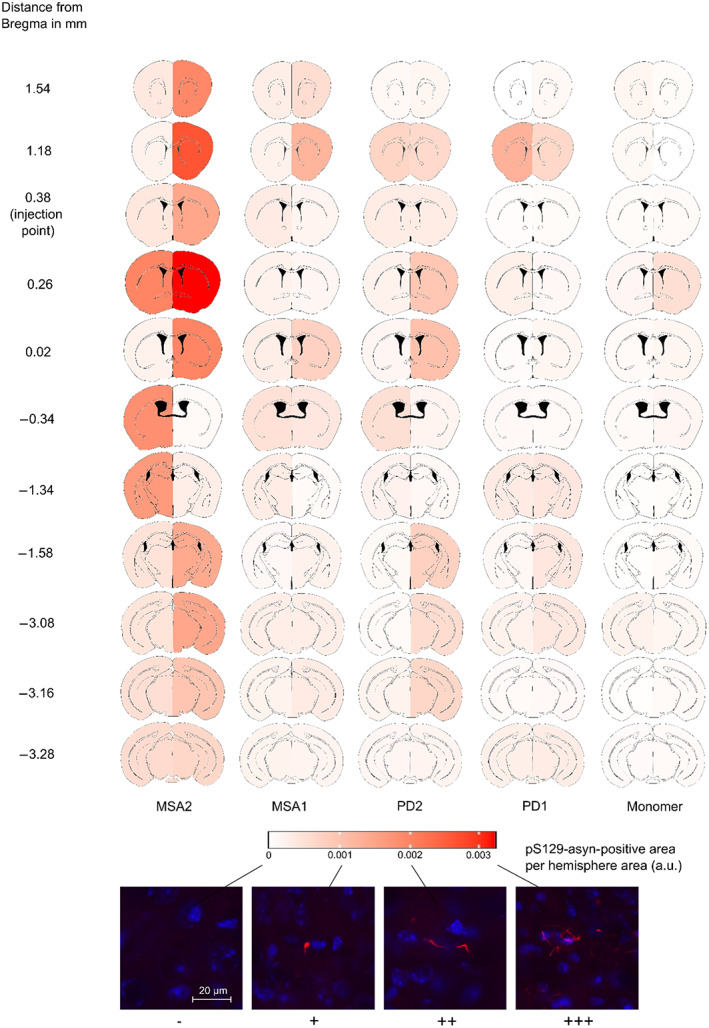
Heatmap showing the distribution of pS129‐positive α‐syn aggregates in the hemispheres of the five treatment groups (MSA2, MSA1, PD2, PD1, α‐syn monomer as control) and the 11 regions throughout the brain ranging from no aggregates (−) to highly abundant in aggregates (+++). Injection point at 0.38 mm relative to Bregma. MSA, multiple system atrophy; PD, Parkinson's disease; α‐syn, alpha‐synuclein.

**FIGURE 4:**
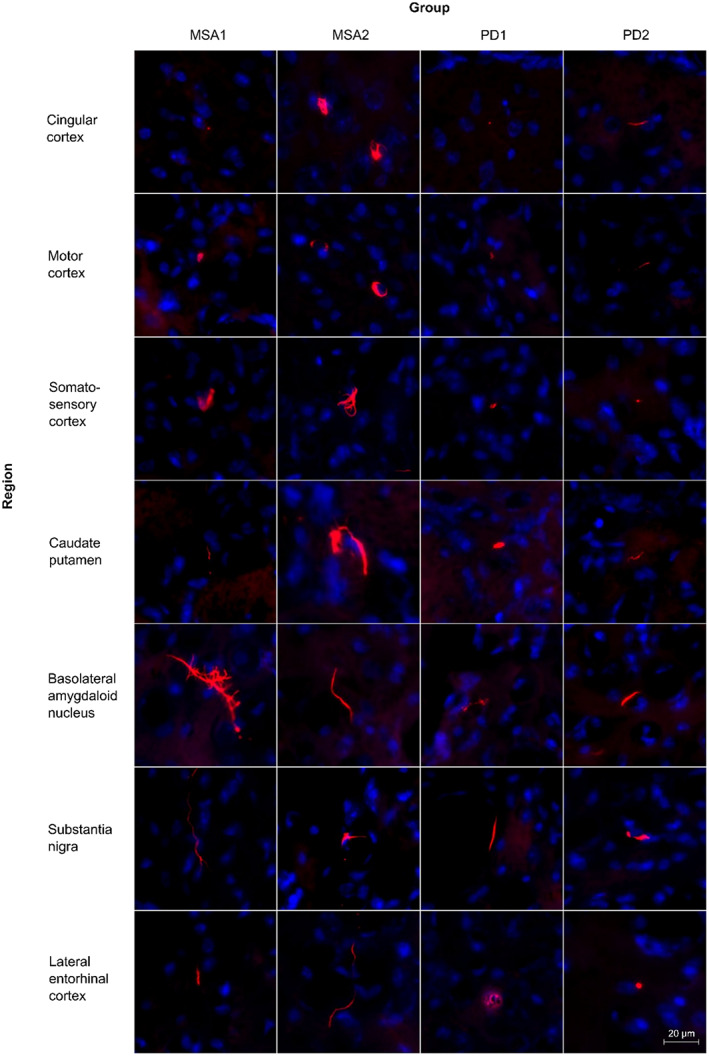
Exemplary images of pS129‐α‐syn aggregates in different brain regions.

Overall, more aggregates were found in the injected than in the non‐injected hemisphere. Brains from the control group injected with α‐syn monomers showed little to no signal with the employed staining method. Furthermore, brains injected with the MSA2‐derived α‐syn fibrils showed the most abundant α‐syn accumulation for both hemispheres and brains from the PD1 group showed the lowest abundance among the fibril‐injected groups (Figure 3 and Supplementary Information Table 1). In the two MSA groups, the pS129‐α‐syn aggregates were also larger and more variable in their morphology (Figure 4).

### 3.2 Strain influences on microglia infiltration

**FIGURE 5:**
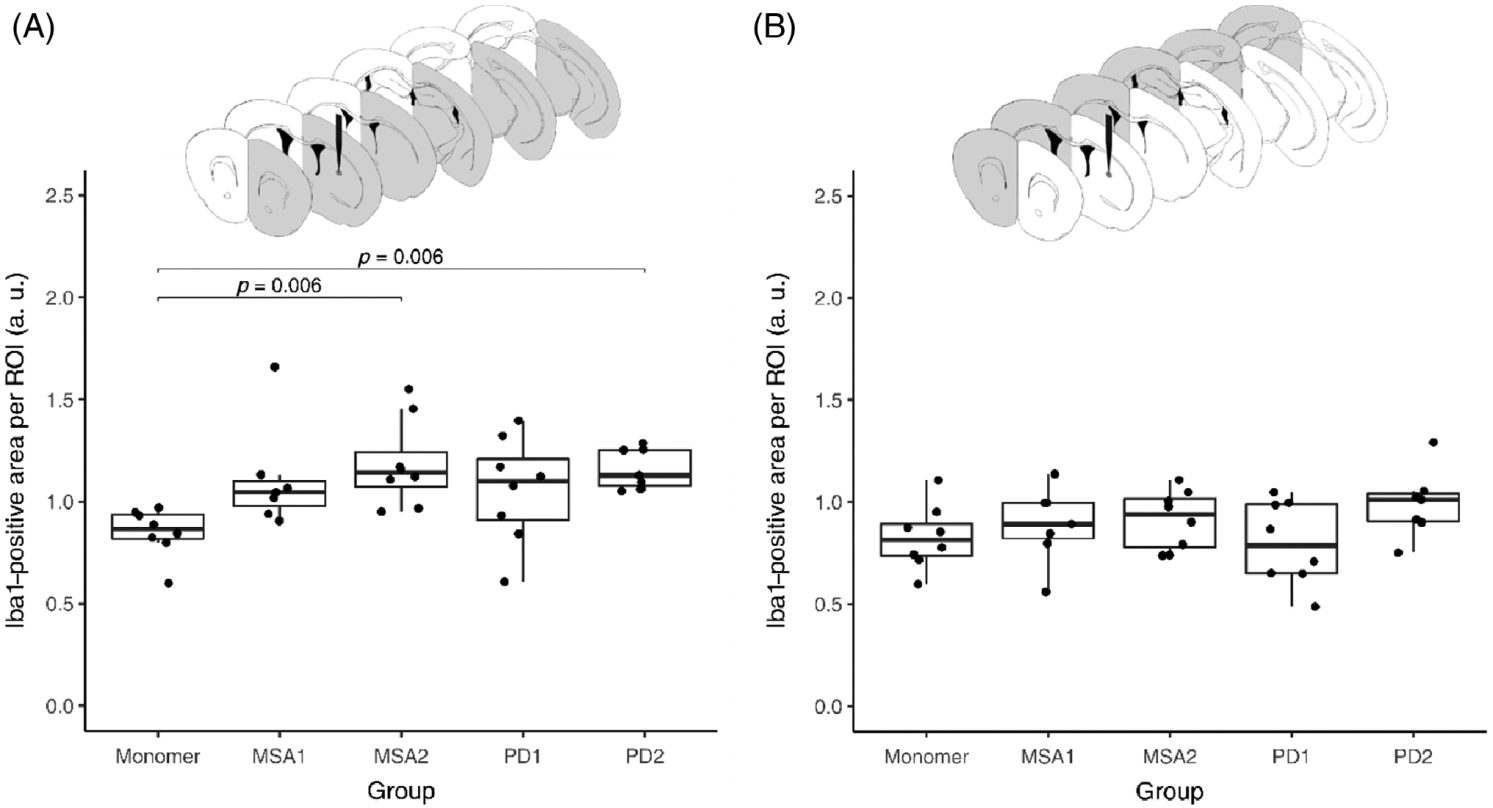
Quantification of Iba1‐positive signal per region of interest (ROI) depending on treatment group for the injected (A) and the non‐injected (B) hemisphere, quantification by area of activated Iba1‐positive microglia divided by the total area of ROI. A *p*‐value <0.05 was considered as significant according to the Kruskal–Wallis test and Dunn's test.

**FIGURE 6:**
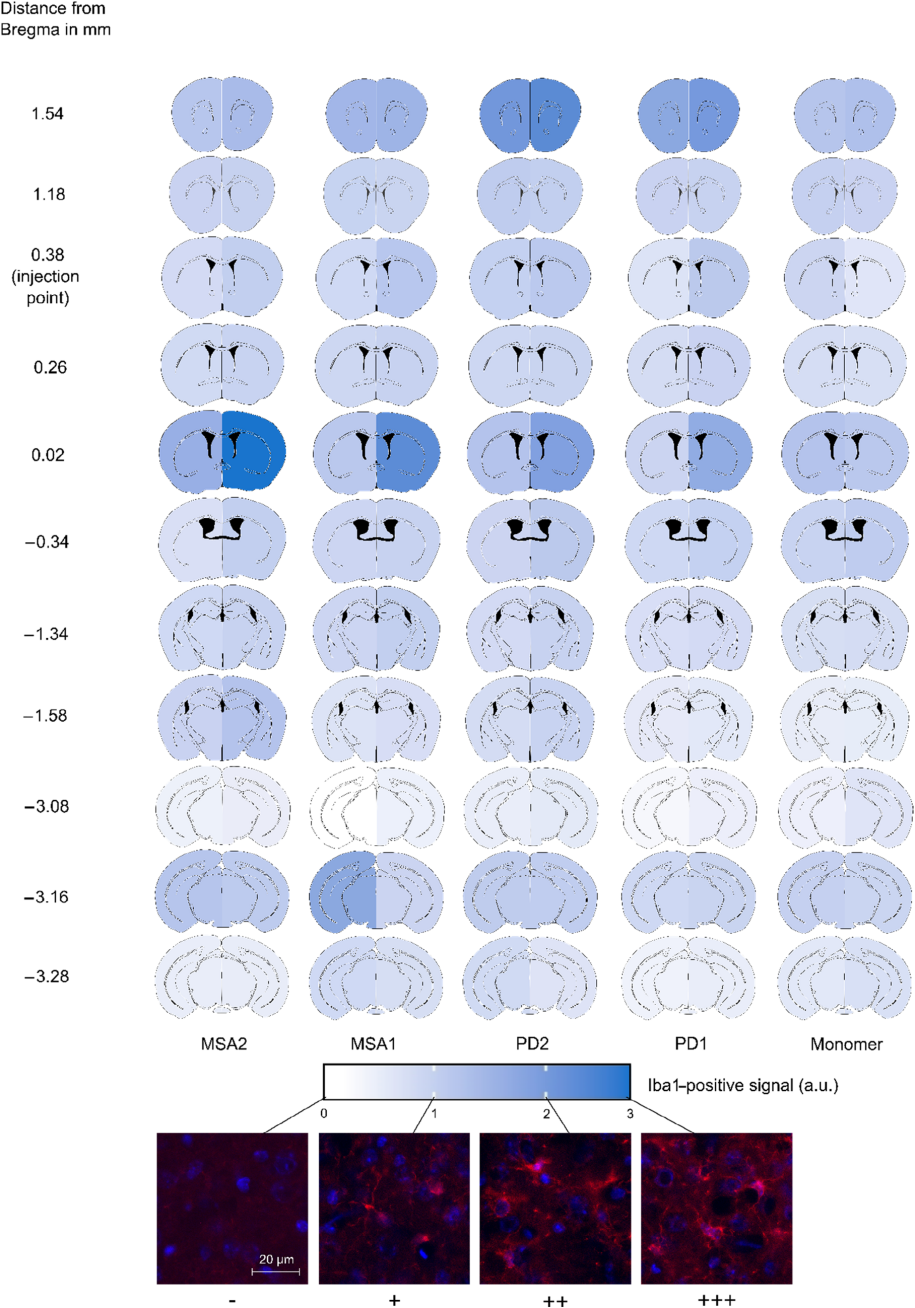
Heatmap showing the distribution of Iba1‐positive signal in the hemispheres of the five treatment groups (MSA2, MSA1, PD2, PD1, α‐syn monomer as control) and the 11 regions throughout the brain ranging from no Iba1‐positive signal (−) to strongest Iba1‐positive signal (+++). Injection point at 0.38 mm relative to Bregma. MSA, multiple system atrophy; PD, Parkinson's disease.

To correlate the microglial response to the pS129‐α‐syn pathology, we quantified the signal of Iba1‐positive microglia in selected sections. Significantly more Iba1‐positive signal was found in the injected hemispheres of MSA2‐ and PD2‐fibril‐injected mice compared to controls (*p* = 0.06, respectively, according to Dunn's test with Benjamini–Hochberg *p*‐value correction; Figure 5A). The Iba1‐positive signal was not significantly different between the groups in the non‐injected hemispheres (Figure 5B). Similar to the α‐syn‐staining, the α‐syn fibril‐injected brains showed Iba1‐signal throughout the entire brain, particularly in the injected hemisphere and in the MSA2‐fibril‐injected brains (Figure 6).

**FIGURE 7:**
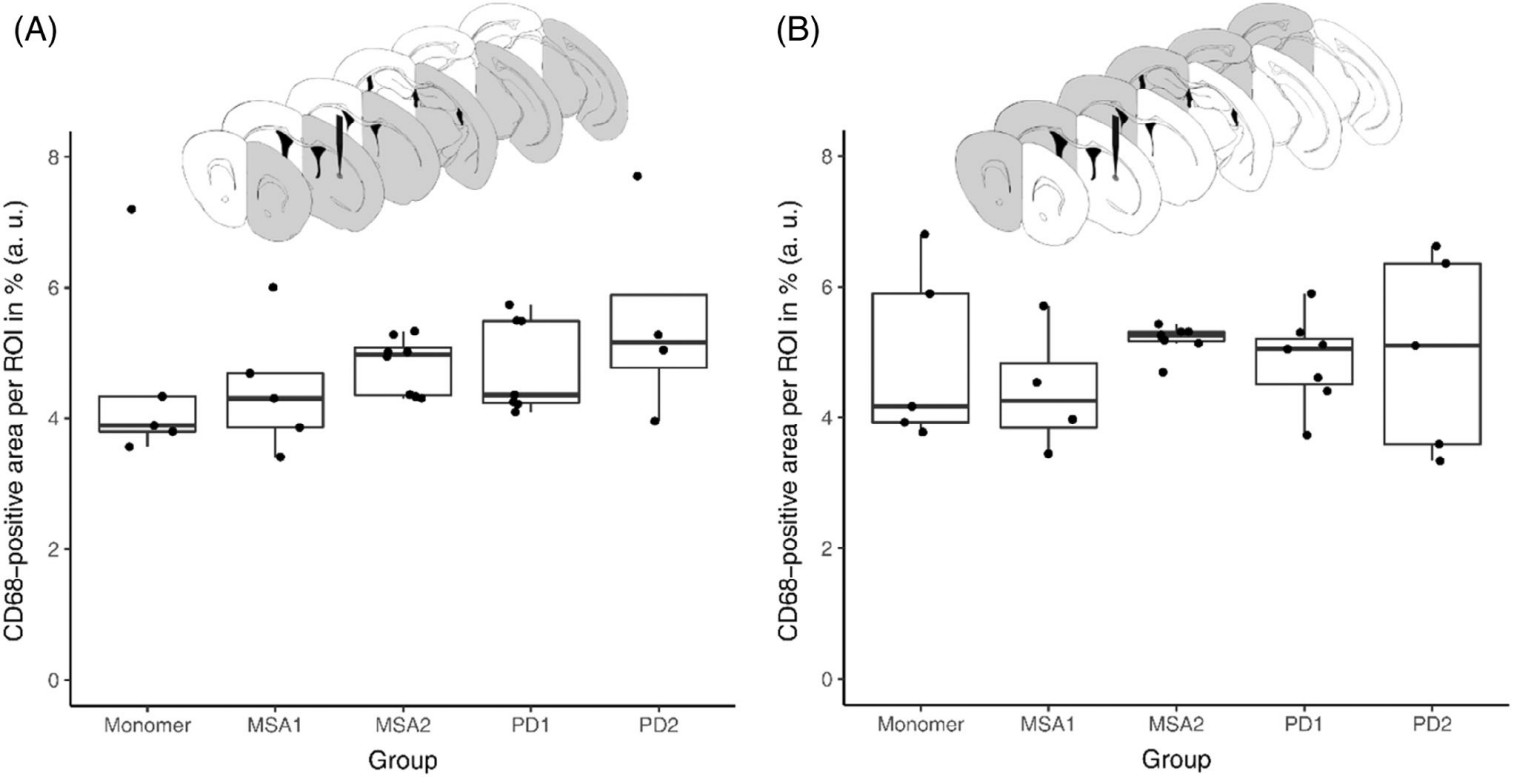
Quantification of CD68‐positive signal per region of interest (ROI) depending on treatment group for the injected (A) and the non‐injected (B) hemisphere, quantification by area of CD68‐positive microglia divided by the total area of ROI.

To assess activated microglia, we also stained for CD68 in two regions (1.18 and 0.26 mm related to Bregma) and analyzed specifically striatal regions where more Iba1‐activation was found than in other regions. Although we did not find significant differences between the treatment groups (injected hemisphere: Kruskal–Wallis chi‐squared = 3.8933, d.f. = 4, *p*‐value = 0.4206; non‐injected hemisphere: Kruskal–Wallis chi‐squared = 2.4669, d.f. = 4, *p*‐value = 0.6506), there was a trend for more CD68‐positive signal particularly in the MSA2 and PD2 groups compared to the monomer group (Figure 7).

### 3.3 Correlation between pS129‐α‐syn pathology and microglia distribution

**FIGURE 8:**
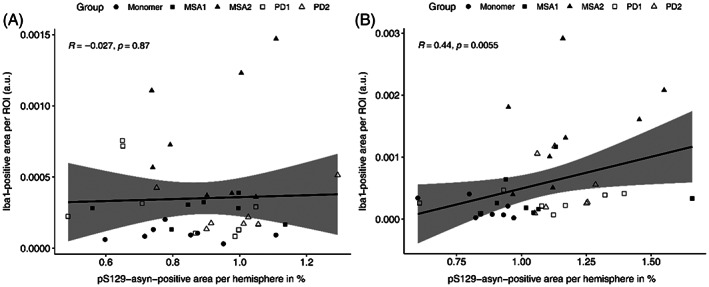
Spearman correlation between pS129‐α‐syn‐positive area per hemisphere in % and area [Iba1]/area [total hemisphere] shown for the injected (A) and non‐injected (B) hemispheres. Treatment groups are indicated by shape.

We then tested for possible correlation between the pS129‐α‐syn pathology and the Iba1‐positive signal in each section. Spearman correlation analysis for the injected hemispheres revealed a weak but significant positive correlation between the two variables (rho = 0.44, *p* = 0.0055; Figure 8A). No significant correlation between pS129‐α‐syn pathology and Iba1‐positive signal was found in the non‐injected hemispheres (rho = −0.027, *p* = 0.87; Figure 8B).

### 3.4 Colocalization of pS129‐α‐syn pathology and CNPase

**FIGURE 9:**
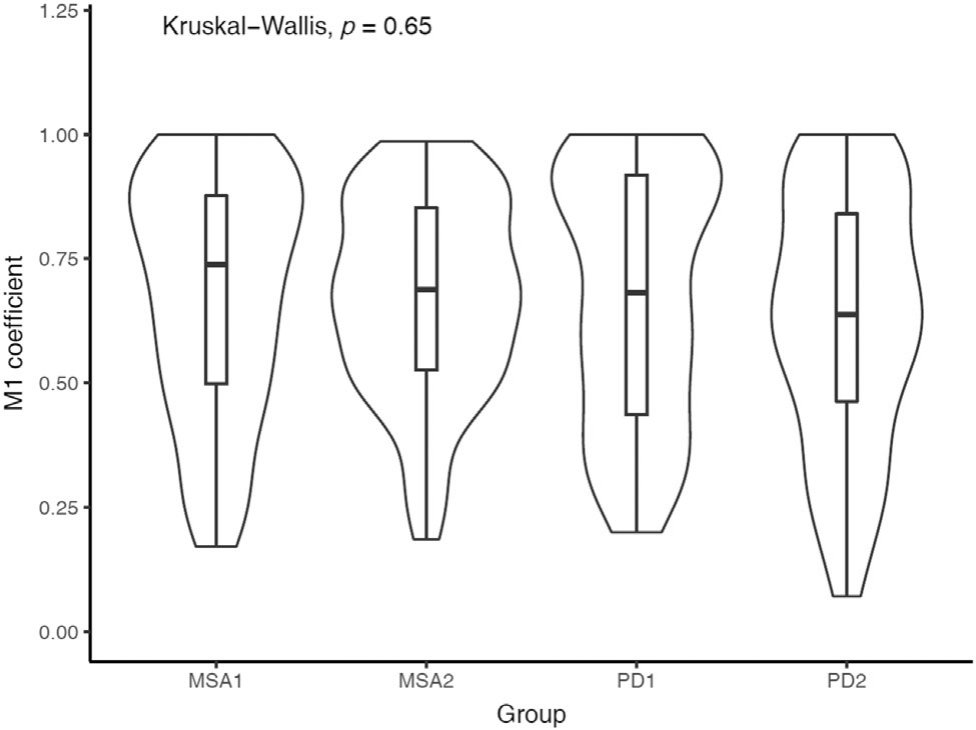
Violin plot for the thresholded M1 coefficient (fraction of pixels in the pS129‐α‐syn channel overlapping with those in the CNPase channel) in the MSA‐fibril‐ and PD‐fibril‐injected groups. CNPase, 2′,3′‐cyclic nucleotide‐3′‐phosphodiesterase; MSA, multiple system atrophy; PD, Parkinson's disease.

In MSA, α‐syn pathology is mostly localized in oligodendrocytes as GCI [2]. Therefore, we performed CNPase staining to mark oligodendrocytes, as well as pS129‐α‐syn staining to examine whether the α‐syn aggregates in the MSA‐fibril‐injected brains are more localized in CNPase‐positive areas than in PD‐fibril‐injected brains. The thresholded Manders' coefficient M1, which describes the fraction of the pixels in the α‐syn channel overlapping with those in the CNPase channel, did not differ significantly between the treatment groups (Kruskal–Wallis chi‐squared = 2.101, d.f. = 3, *p*‐value = 0.5517). Nor did the M1 coefficient differ between MSA‐fibril‐ and PD‐fibril‐injected brains in general (Wilcoxon rank‐sum test, *W* = 8992.5, *p*‐value = 0.6464) or between the four groups (Kruskal–Wallis chi‐squared = 1.6389, d.f. = 3, *p*‐value = 0.6506; Figure 9).

### 3.5 Influence of α‐syn injections on astrocytes

**FIGURE 10:**
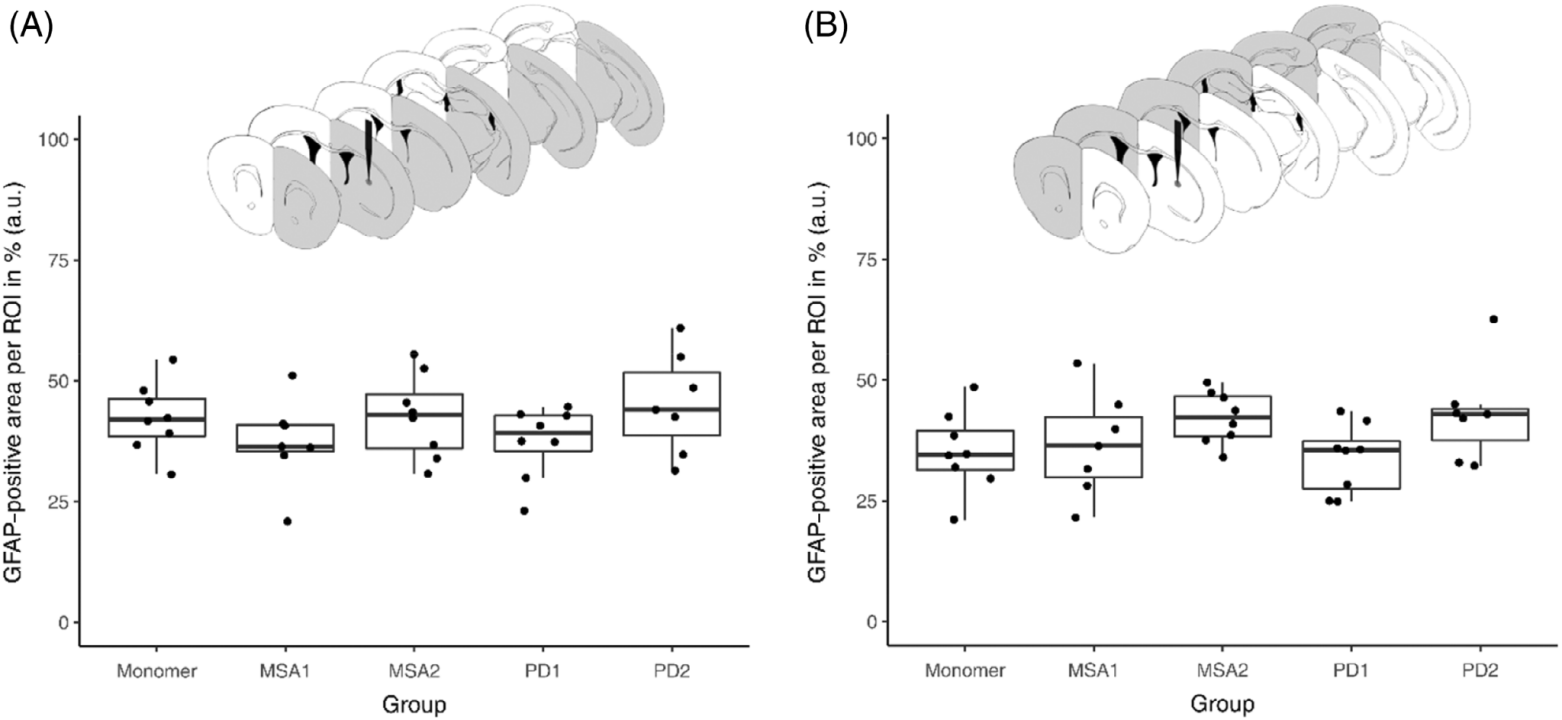
Quantification of GFAP‐positive signal per region of interest (ROI) depending on treatment group for the injected (A) and the non‐injected (B) hemisphere, quantification by area of GFAP‐positive astrocytes divided by the total area of ROI.

We also stained for GFAP in five different brain regions (+1.18, +0.26, − 0.34, − 1.34, and −3.16 mm related to Bregma) to analyze any influence of α‐syn‐fibril injection on astrocytes. For all five groups and both hemispheres, we did not detect any significant differences in the GFAP‐positive area per ROI (ANOVA of injected hemispheres, *p* = 0.313; ANOVA of non‐injected hemispheres, *p* = 0.132; Figure 10).

### 3.6 Influence of α‐syn injections on dopaminergic neuron survival

To assess the impact of α‐syn‐fibril or α‐syn‐monomer injections on the survival of dopaminergic neurons in the SNpc, we performed immunohistochemical labeling for TH and quantified the number of dopaminergic neurons. There were no significant differences in the number of TH‐positive cells in the SNpc between the five groups in either of the hemispheres (ANOVA of injected hemispheres, *p* = 0.365; ANOVA of non‐injected hemispheres, *p* = 0.681).

## 4 DISCUSSION

Although neurodegenerative movement disorders such as PD and MSA are α‐synucleinopathies with similar disease mechanisms, clinical disease phenotypes often vary between individuals, even within the same disease entity. Previous reports postulated that different α‐syn strains may cause different phenotypes in PD and MSA patients and, next to genetic background and environmental influences, can be one reason for interindividual differences in patients suffering from the same disease [24]. To model the seeding and spreading of α‐syn aggregates, we injected α‐syn fibrils amplified from two PD and two MSA patient brain extracts into mouse brains. These fibrils did not have defined molecular structures for one type of disease but rather expressed a pronounced diversity [13], which is being reflected by the different levels of α‐syn pathology in our mouse model. In addition, the amplified fibrils were structurally different to non‐brain‐derived α‐syn fibrils and might be a more accurate model to study α‐synucleinopathies [13].

As previously reported, seeding of α‐syn preformed fibrils (PFFs), which were aggregated in the absence of brain‐derived seeds, induce spreading of α‐syn pathology [5]. Similar to previous studies, where PFF strains of MSA patients more potently induced α‐syn‐spreading pathology than those of PD patients [24], the most pronounced α‐syn‐spreading in our study was observed in animals injected with α‐syn fibrils amplified from the brain homogenate of one MSA patient (Figure 3). Of all patients studied, this patient (MSA2) also had the shortest disease duration of only 6 years and died the youngest at an age of 71, suggesting a more aggressive disease course than patient MSA1, whose disease duration was longer (7 years) and who died at an older age (82 years; Table 1). The fibrils of the two PD patients, on the other hand, were less potent in seeding α‐syn‐pathology compared to MSA2, with PD2 and MSA1‐induced pS129‐positive α‐syn pathology being comparable. All brain‐derived α‐syn fibrils induced more α‐syn aggregates than the group of α‐syn monomers. Our finding that strong α‐syn spreading was induced in the brains injected with α‐syn fibrils amplified from MSA patient brain homogenate is in accordance with the rapidly progressive disease course and overall poor prognosis of MSA compared to PD. However, MSA1 and MSA2 showed marked differences regarding α‐syn distribution, suggesting additional individual factors contributing to spreading besides the disease entity.

In our study, we detected pS129‐α‐syn‐positive aggregates throughout the whole brain, although the non‐injected hemisphere was less affected. Notably, in our study, the injection site at Bregma 0.38 mm did not show the strongest pS129‐α‐syn pathology (Figure 3), suggesting that α‐syn pathology has spread to brain regions connected to the injection site. Consistent with this observation, previous studies demonstrated that α‐syn aggregates propagate through neuronal networks [25, 26, 27]. In addition, LB‐like inclusions after striatal injection of recombinant α‐syn‐fibrils can be found in several areas innervating the striatum including the frontal and insular cortices, the amygdala, and the SNpc [6, 26]. Interhemispheric corticostriatal and nigrostriatal connections have also been described [28] through which the propagation might occur.

Previous studies indicated that different α‐syn strains have distinct levels of toxicity, seeding, and propagation properties [23, 29]. Recombinant and brain‐derived α‐syn strains, for instance, differ in their clinical and pathological manifestations after propagation in a mouse model [30]. This includes variable disease incubation periods, distinct regional and cellular vulnerability to α‐syn aggregates and conformational differences of these aggregates. Structural differences in individual strains, as demonstrated by different protonation levels of specific residues [13] may be responsible for their different pathological properties. In contrast to our study, strains were often either generated from the same precursor α‐syn [9] or were generated de novo [10] and not seeded from different patient brains as in our study. In addition, fibrils from the same species are known to induce more pathology than cross‐species seeded fibrils from humans to mice and vice versa [27, 31, 32].

Increasing evidence suggests a relationship between α‐syn pathology and immune response activation in PD [33, 34, 35, 36]. Inflammatory responses, including microglia and astrocyte activation, infiltration of peripheral immune cells, and alterations of leukocytes in the spleen and lymph nodes occur after intrastriatal injection of α‐syn PFFs [37]. We, therefore, studied the amount of microglial infiltration after injection of different brain‐derived α‐syn fibrils. Similar to the pS129‐α‐syn‐staining, brains from the MSA2‐fibril‐injected group showed the highest abundance of Iba1‐positive signal. Further statistical analysis supported a correlation between α‐syn‐pathology and microglia abundance for the injected hemisphere.

Increased microglial activation has also been detected in positron emission tomography (PET) scans of patients with α‐synucleinopathies [38]. For example, patients with early‐stage parkinsonian phenotype of MSA (MSA‐P) showed widespread microglia activation [39]. In addition, patients with idiopathic rapid‐eye‐movement sleep behavior disorder had increased microglial activation in the SN witnessed by ^11^C‐PK11195‐PET along with reduced dopaminergic function in the putamen [38]. However, it is yet unresolved how microglia are activated by α‐syn aggregates and whether their activation contributes to the formation of these aggregates. Duffy et al. [40] proposed that microglial activation might be partly responsible for neuronal degeneration in the SNpc. A contribution of microglia to the propagation of α‐syn pathology was also suggested from recent work in our lab using iron‐treated mice [5]. Other reports, on the other hand, favor a neuroprotective function of microglia as they clear α‐syn released from neurons [41].

We did not find significant astrogliosis for any of the treatment groups in accordance to postmortem MSA patient brain analyses, which also did not find reactive astrogliosis but widespread microgliosis especially in the white matter [42]. For PD, studies have reported reactive astrogliosis in the SN [43, 44, 45]. These results were not reproduced in our study, most likely because our model represents an earlier disease stage without any dopaminergic cell loss in the SNpc.

## 5 CONCLUSION

Our study shows that α‐syn fibrils amplified from the brain homogenates of different MSA and PD patients differ in their capacity to seed α‐syn pathology and to induce microglial activation after intrastriatal injection into mouse brains. We further find that the strongest α‐syn pathology was triggered by α‐syn fibrils amplified from one of the two MSA patient brain homogenates in agreement with previous studies indicating more efficient spreading and a more rapid disease course for MSA when compared to PD. Individual factors are likely to contribute to differential seeding ability beyond the disease entity itself. A similar heterogeneity has also been observed in the amplification of α‐syn fibrils out of cerebrospinal fluid from different MSA/PD patients [46]. The current study forms the basis for future investigations with larger patient numbers and more human‐like models to gain insight into the heterogeneity of patient‐derived α‐syn fibrils and the importance of α‐syn spreading for the course of α‐synucleinopathy diseases.

## AUTHOR CONTRIBUTIONS

Shuyu Zhang, Karina Dauer, Lars Tatenhorst, Lucas Caldi Gomes, Friederike Liesche‐Starnecker, Timo Strohäker, Markus Zweckstetter, and Paul Lingor planned and designed the experiments. Shuyu Zhang, Karina Dauer, Timo Strohäker, Simon Mayer, and Lars Tatenhorst performed the experiments. Byung Chul Jung and Seung‐Jae Lee performed brain extract preparation and PMCA. Woojin S. Kim organized patient brain samples. Stefan Becker prepared monomeric α‐syn. Timo Strohäker and Markus Zweckstetter generated the patient‐derived α‐syn fibrils. Shuyu Zhang and Paul Lingor performed statistical analyses of the study and interpreted the data. Paul Lingor supervised the study. Shuyu Zhang, Lars Tatenhorst, Lucas Caldi Gomes, Markus Zweckstetter, and Paul Lingor wrote the manuscript and designed the figures.

## CONFLICT OF INTEREST STATEMENT

The authors declare no conflict of interests.

## ETHICS STATEMENT

Ethics approval for the study of brain tissue was from the University of New South Wales Human Research Ethics Committee (approval number: HC16568). The animals were treated according to the EU Directive 2010/63/EU for animal experiments and the regulations of the local animal research council as well as the legislation of the State of Lower Saxony, Germany (ethics approval number: 33.9‐42502‐04‐15/1982) in an exploratory study which has received approval from the institutional ethics committee.

## Supporting information


**Data S1.** Supporting Information.

## Data Availability

The datasets used and analyzed during the current study are available from the corresponding author on reasonable request.

## References

[1] Videnovic A . Management of sleep disorders in Parkinson's disease and multiple system atrophy. Mov Disord. 2017;32(5):659–668. 28116784 10.1002/mds.26918 PMC5435534

[2] Papp MI , Lantos PL . The distribution of oligodendroglial inclusions in multiple system atrophy and its relevance to clinical symptomatology. Brain. 1994;117(Pt 2):235–243. 8186951 10.1093/brain/117.2.235

[3] Polinski NK . A summary of phenotypes observed in the In vivo rodent alpha‐Synuclein preformed fibril model. J Parkinsons Dis. 2021;11(4):1555–1567. 34486988 10.3233/JPD-212847 PMC8609716

[4] Recasens A , Ulusoy A , Kahle PJ , Di Monte DA , Dehay B . In vivo models of alpha‐synuclein transmission and propagation. Cell Tissue Res. 2018;373(1):183–193. 29185072 10.1007/s00441-017-2730-9

[5] Dauer Née Joppe K , Tatenhorst L , Caldi Gomes L , Zhang S , Parvaz M , Carboni E , et al. Brain iron enrichment attenuates α‐synuclein spreading after injection of preformed fibrils. J Neurochem. 2021;159(3):554–573. 34176164 10.1111/jnc.15461

[6] Paumier KL , Luk KC , Manfredsson FP , Kanaan NM , Lipton JW , Collier TJ , et al. Intrastriatal injection of pre‐formed mouse α‐synuclein fibrils into rats triggers α‐synuclein pathology and bilateral nigrostriatal degeneration. Neurobiol Dis. 2015;82:185–199. 26093169 10.1016/j.nbd.2015.06.003 PMC4640952

[7] Recasens A , Dehay B , Bové J , Carballo‐Carbajal I , Dovero S , Pérez‐Villalba A , et al. Lewy body extracts from Parkinson disease brains trigger α‐synuclein pathology and neurodegeneration in mice and monkeys. Ann Neurol. 2014;75(3):351–362. 24243558 10.1002/ana.24066

[8] Luk KC , Kehm VM , Zhang B , O'Brien P , Trojanowski JQ , Lee VMY . Intracerebral inoculation of pathological α‐synuclein initiates a rapidly progressive neurodegenerative α‐synucleinopathy in mice. J Exp Med. 2012;209(5):975–986. 22508839 10.1084/jem.20112457 PMC3348112

[9] Bousset L , Pieri L , Ruiz‐Arlandis G , Gath J , Jensen PH , Habenstein B , et al. Structural and functional characterization of two alpha‐synuclein strains. Nat Commun. 2013;4(1):2575. 24108358 10.1038/ncomms3575 PMC3826637

[10] Rey NL , Bousset L , George S , Madaj Z , Meyerdirk L , Schulz E , et al. α‐Synuclein conformational strains spread, seed and target neuronal cells differentially after injection into the olfactory bulb. Acta Neuropathol Commun. 2019;7(1):221. 31888771 10.1186/s40478-019-0859-3 PMC6937797

[11] Woerman AL , Stöhr J , Aoyagi A , Rampersaud R , Krejciova Z , Watts JC , et al. Propagation of prions causing synucleinopathies in cultured cells. Proc Natl Acad Sci USA. 2015;112(35):E4949–E4958. 26286986 10.1073/pnas.1513426112 PMC4568231

[12] Prusiner SB , Woerman AL , Mordes DA , Watts JC , Rampersaud R , Berry DB , et al. Evidence for α‐synuclein prions causing multiple system atrophy in humans with parkinsonism. Proc Natl Acad Sci USA. 2015;112(38):E5308–E5317. 26324905 10.1073/pnas.1514475112 PMC4586853

[13] Strohäker T , Jung BC , Liou S‐H , Fernandez CO , Riedel D , Becker S , et al. Structural heterogeneity of α‐synuclein fibrils amplified from patient brain extracts. Nat Commun. 2019;10(1):5535. 31797870 10.1038/s41467-019-13564-w PMC6893031

[14] Johnson M, Coulton AT, Geeves MA, Mulvihill DP. Targeted amino‐terminal acetylation of recombinant proteins in E. coli. PLoS ONE. 2010;5(12):e15801. 21203426 10.1371/journal.pone.0015801 PMC3009751

[15] Hoyer W , Antony T , Cherny D , Heim G , Jovin TM , Subramaniam V . Dependence of α‐synuclein aggregate morphology on solution conditions. J Mol Biol. 2002;322(2):383–393. 12217698 10.1016/s0022-2836(02)00775-1

[16] Paxinos G , Franklin K . Paxinos and Franklin's the mouse brain in stereotaxic coordinates. London: Elsevier Science; 2019. Available from: https://books.google.de/books?id=x3aQDwAAQBAJ

[17] Schindelin J , Arganda‐Carreras I , Frise E , Kaynig V , Longair M , Pietzsch T , et al. Fiji: an open‐source platform for biological‐image analysis. Nat Methods. 2012;9(7):676–682. 22743772 10.1038/nmeth.2019 PMC3855844

[18] Bolte S , Cordelières FP . A guided tour into subcellular colocalization analysis in light microscopy. J Microsc. 2006;224(Pt 3):213–232. 17210054 10.1111/j.1365-2818.2006.01706.x

[19] Berg S , Kutra D , Kroeger T , Straehle CN , Kausler BX , Haubold C , et al. ilastik: interactive machine learning for (bio)image analysis. Nat Methods. 2019;16(12):1226–1232. 31570887 10.1038/s41592-019-0582-9

[20] Luk KC , Kehm V , Carroll J , Zhang B , O'Brien P , Trojanowski JQ , et al. Pathological α‐synuclein transmission initiates Parkinson‐like neurodegeneration in nontransgenic mice. Science (New York, N.Y.). 2012;338(6109):949–953. Available from: https://pubmed.ncbi.nlm.nih.gov/23161999/ 23161999 10.1126/science.1227157 PMC3552321

[21] Thomsen MB , Ferreira SA , Schacht AC , Jacobsen J , Simonsen M , Betzer C , et al. PET imaging reveals early and progressive dopaminergic deficits after intra‐striatal injection of preformed alpha‐synuclein fibrils in rats. Neurobiol Dis. 2021;149:105229. 33352233 10.1016/j.nbd.2020.105229

[22] Alam P , Bousset L , Melki R , Otzen DE . α‐Synuclein oligomers and fibrils: a spectrum of species, a spectrum of toxicities. J Neurochem. 2019;150(5):522–534. 31254394 10.1111/jnc.14808

[23] Peelaerts W , Bousset L , van der Perren A , Moskalyuk A , Pulizzi R , Giugliano M , et al. α‐Synuclein strains cause distinct synucleinopathies after local and systemic administration. Nature. 2015;522(7556):340–344. 26061766 10.1038/nature14547

[24] van der Perren A , Gelders G , Fenyi A , Bousset L , Brito F , Peelaerts W , et al. The structural differences between patient‐derived α‐synuclein strains dictate characteristics of Parkinson's disease, multiple system atrophy and dementia with Lewy bodies. Acta Neuropathol. 2020;139(6):977–1000. 32356200 10.1007/s00401-020-02157-3 PMC7244622

[25] Chung HK , Ho H‐A , Pérez‐Acuña D , Lee S‐J . Modeling α‐synuclein propagation with preformed fibril injections. J Mov Disord. 2019;12(3):139–151. 31556259 10.14802/jmd.19046 PMC6763716

[26] Masuda‐Suzukake M , Nonaka T , Hosokawa M , Kubo M , Shimozawa A , Akiyama H , et al. Pathological alpha‐synuclein propagates through neural networks. Acta Neuropathol Commun. 2014;2:88. 25095794 10.1186/s40478-014-0088-8 PMC4147188

[27] Rey NL , George S , Steiner JA , Madaj Z , Luk KC , Trojanowski JQ , et al. Spread of aggregates after olfactory bulb injection of α‐synuclein fibrils is associated with early neuronal loss and is reduced long term. Acta Neuropathol. 2018;135(1):65–83. 29209768 10.1007/s00401-017-1792-9 PMC5756266

[28] Lieu CA , Subramanian T . The interhemispheric connections of the striatum: implications for Parkinson's disease and drug‐induced dyskinesias. Brain Res Bull. 2012;87(1):1–9. 21963946 10.1016/j.brainresbull.2011.09.013 PMC3246032

[29] Peng C , Gathagan RJ , Covell DJ , Medellin C , Stieber A , Robinson JL , et al. Cellular milieu imparts distinct pathological α‐synuclein strains in α‐synucleinopathies. Nature. 2018;557(7706):558–563. 29743672 10.1038/s41586-018-0104-4 PMC5970994

[30] Lau A , So RWL , Lau HHC , Sang JC , Ruiz‐Riquelme A , Fleck SC , et al. α‐Synuclein strains target distinct brain regions and cell types. Nat Neurosci. 2020;23(1):21–31. 31792467 10.1038/s41593-019-0541-x PMC6930851

[31] Fares M‐B , Maco B , Oueslati A , Rockenstein E , Ninkina N , Buchman VL , et al. Induction of de novo α‐synuclein fibrillization in a neuronal model for Parkinson's disease. Proc Natl Acad Sci USA. 2016;113(7):E912–E921. 26839406 10.1073/pnas.1512876113 PMC4763739

[32] Luk KC , Covell DJ , Kehm VM , Zhang B , Song IY , Byrne MD , et al. Molecular and biological compatibility with host alpha‐synuclein influences fibril pathogenicity. Cell Rep. 2016;16(12):3373–3387. 27653697 10.1016/j.celrep.2016.08.053 PMC5087609

[33] Caldi Gomes L , Galhoz A , Jain G , Roser A‐E , Maass F , Carboni E , et al. Multi‐omic landscaping of human midbrains identifies disease‐relevant molecular targets and pathways in advanced‐stage Parkinson's disease. Clin Transl Med. 2022;12(1):e692. 35090094 10.1002/ctm2.692 PMC8797064

[34] Kannarkat GT , Boss JM , Tansey MG . The role of innate and adaptive immunity in Parkinson's disease. J Parkinsons Dis. 2013;3(4):493–514. 24275605 10.3233/JPD-130250 PMC4102262

[35] Schonhoff AM , Williams GP , Wallen ZD , Standaert DG , Harms AS . Innate and adaptive immune responses in Parkinson's disease. Prog Brain Res. 2020;252:169–216. 32247364 10.1016/bs.pbr.2019.10.006 PMC7185735

[36] Tansey MG , Romero‐Ramos M . Immune system responses in Parkinson's disease: early and dynamic. Eur J Neurosci. 2019;49(3):364–383. 30474172 10.1111/ejn.14290 PMC6391192

[37] Earls RH , Menees KB , Chung J , Barber J , Gutekunst C‐A , Hazim MG , et al. Intrastriatal injection of preformed alpha‐synuclein fibrils alters central and peripheral immune cell profiles in non‐transgenic mice. J Neuroinflammation. 2019;16(1):250. 31796095 10.1186/s12974-019-1636-8 PMC6889316

[38] Stokholm MG , Iranzo A , Østergaard K , Serradell M , Otto M , Svendsen KB , et al. Assessment of neuroinflammation in patients with idiopathic rapid‐eye‐movement sleep behaviour disorder: a case–control study. Lancet Neurol. 2017;16(10):789–796. 28684245 10.1016/S1474-4422(17)30173-4

[39] Kübler D , Wächter T , Cabanel N , Su Z , Turkheimer FE , Dodel R , et al. Widespread microglial activation in multiple system atrophy. Mov Disord. 2019;34(4):564–568. 30726574 10.1002/mds.27620 PMC6659386

[40] Duffy MF , Collier TJ , Patterson JR , Kemp CJ , Fischer DL , Stoll AC , et al. Quality over quantity: advantages of using alpha‐Synuclein preformed fibril triggered synucleinopathy to model idiopathic Parkinson's disease. Front Neurosci. 2018;12:621. 30233303 10.3389/fnins.2018.00621 PMC6132025

[41] Choi I , Zhang Y , Seegobin SP , Pruvost M , Wang Q , Purtell K , et al. Microglia clear neuron‐released α‐synuclein via selective autophagy and prevent neurodegeneration. Nat Commun. 2020;11(1):1386. 32170061 10.1038/s41467-020-15119-w PMC7069981

[42] Nykjaer CH , Brudek T , Salvesen L , Pakkenberg B . Changes in the cell population in brain white matter in multiple system atrophy. Mov Disord. 2017;32(7):1074–1082. 28394027 10.1002/mds.26979

[43] Thannickal TC , Lai Y‐Y , Siegel JM . Hypocretin (orexin) cell loss in Parkinson's disease. Brain. 2007;130(Pt 6):1586–1595. 17491094 10.1093/brain/awm097 PMC8762453

[44] Lastres‐Becker I , Ulusoy A , Innamorato NG , Sahin G , Rábano A , Kirik D , et al. α‐Synuclein expression and Nrf2 deficiency cooperate to aggravate protein aggregation, neuronal death and inflammation in early‐stage Parkinson's disease. Hum Mol Genet. 2012;21(14):3173–3192. 22513881 10.1093/hmg/dds143

[45] Renkawek K , Stege GJ , Bosman GJ . Dementia, gliosis and expression of the small heat shock proteins hsp27 and alpha B‐crystallin in Parkinson's disease. Neuroreport. 1999;10(11):2273–2276. 10439447 10.1097/00001756-199908020-00009

[46] Shahnawaz M , Mukherjee A , Pritzkow S , Mendez N , Rabadia P , Liu X , et al. Discriminating α‐synuclein strains in Parkinson's disease and multiple system atrophy. Nature. 2020;578(7794):273–277. 32025029 10.1038/s41586-020-1984-7 PMC7066875

