# Supplementary material for: Alpha‐synuclein fibrils amplified from multiple system atrophy and Parkinson's disease patient brain spread after intracerebral injection into mouse brain
Source: Brain Pathol. 2023 Jul 24;33(5):e13196. doi: 10.1111/bpa.13196 (PMC10467043; doi:10.1111/bpa.13196)
Supplement: Supplementary file 1 — Data S1. Supporting Information. [file BPA-33-e13196-s001.pdf]

	pS129-α-syn left		pS129-α-syn right		Iba1 left		Iba1 right	
**Group**	Mean	SD	Mean	SD	Mean	SD	Mean	SD
**Monomer**	6.98E-06	1.30E-05	6.25E-05	1.10E-04	0.8100272	0.2143593	0.8404325	0.1375097
**MSA1**	1.49E-04	1.15E-04	1.46E-04	1.00E-04	0.8478938	0.1547692	1.1714338	0.3616426
**MSA2**	3.67E-04	2.90E-04	1.15E-03	8.10E-04	0.9182369	0.1820765	1.2399634	0.2880812
**PD1**	7.88E-05	8.78E-05	1.10E-04	9.38E-05	0.7808669	0.2834459	1.0809529	0.3190348
**PD2**	8.25E-05	5.99E-05	2.09E-04	2.02E-04	1.0414402	0.2145663	1.2240484	0.1489991

Supplementary Table 1: Quantification of immunofluorescence for pS129-α-syn and the Iba1 staining. Data represents signal-positive area/total ROI area and is given as mean and standard deviation [SD].

Staining	Regions stained related to Bregma	ROIs	Magnification	Number of sections analyzed
**anti-pS129 α-syn (EP1536Y)**	+ 1.54 mm, + 1.18 mm, + 0.38 mm, + 0.26 mm, + 0.02 mm, - 0.34 mm, - 1.34 mm, - 1.58 mm, - 3.08 mm, - 3.16 mm, - 3.28 mm	Right and left hemisphere	10x	392 (MSA1 = 70, MSA2 = 86, PD1 = 87, PD2 = 71, Monomer = 78)
**anti-Iba1 (019-19741)**	+ 1.54 mm, + 0.38 mm, + 0.02 mm, - 1.58 mm, - 3.08 mm, - 3.28 mm	3 circular ROIs per hemisphere	10x	221 (MSA1 = 41, MSA2 = 47, PD1 = 47, PD2 = 41, Monomer = 45)
**anti-Iba1 (011-27991)**	+ 1.18 mm, + 0.26 mm, - 0.34 mm, - 1.34 mm, - 3.16 mm	3 circular ROIs per hemisphere	10x	85 (MSA1 = 14, MSA2 = 18, PD1 = 19, PD2 = 16, Monomer = 18)
**anti-CD68 (FA-11, ab53444)**	+ 1.18 mm, + 0.26 mm	1 circular ROI per hemisphere	10x	40 (MSA1 = 6, MSA2 = 11, PD1 = 10, PD2 = 5, Monomer = 8)
**anti-pS129 α-syn (EP1536Y)/ anti-CNPase (ab6319)**	+ 0.20 mm	Systematic meandering through section, image taken when α-syn positive signal was detected	63x	30 (MSA1 = 7, MSA2 = 8, PD1 = 8, PD2 = 7) (264 images)
**anti-GFAP (13-0300)**	+ 1.18 mm, + 0.26 mm, - 0.34 mm, - 1.34 mm, - 3.16 mm	3 circular ROIs per hemisphere	10x	166 (MSA1 = 28, MSA2 = 38, PD1 = 39, PD2 = 30, Monomer = 31)
**anti-TH (620-0336)**	+ 3.00 mm, + 3.15 mm, + 3.30 mm	Right and left SNpc	5x	114 (MSA1 = 21, MSA2 = 24, PD1 = 21, PD2 = 24, Monomer = 24)

Supplementary Table 2: Overview of regions stained related to Bregma, regions of interest (ROIs), magnification, and number of sections analyzed for each staining, respectively.
